# Epithelioid Trophoblastic Tumor Similar to Uterine Arteriovenous Malformation: A Case Report and Literature Review

**DOI:** 10.1002/ccr3.72264

**Published:** 2026-03-17

**Authors:** Huihua Xiang, Xiaoxiao Feng, Shanrong Shu

**Affiliations:** ^1^ Department of Gynecology The First Affiliated Hospital of Jinan University Guangzhou China

**Keywords:** epithelioid trophoblastic tumor, hypervascular cervical mass, uterine arteriovenous malformation, uterine artery embolization, vaginal hemorrhage

## Abstract

Epithelioid trophoblastic tumor (ETT) is a rare subtype of gestational trophoblastic neoplasia, most commonly arising in the cervix or lower uterine segment and often associated with mildly elevated serum human chorionic gonadotropin (hCG). Owing to its nonspecific clinical and imaging features, preoperative diagnosis remains challenging, particularly when the tumor exhibits hypervascular characteristics mimicking uterine arteriovenous malformation (AVM). We report the case of a 48‐year‐old perimenopausal woman who presented with sudden‐onset massive vaginal bleeding following a short period of amenorrhea. Transvaginal color Doppler ultrasonography and contrast‐enhanced computed tomography revealed a hypervascular cervical mass with multiple enlarged and tortuous vessels, raising strong suspicion of uterine AVM. Given the high risk of hemorrhage, preoperative uterine artery embolization was performed, followed by laparoscopic total hysterectomy with bilateral salpingo‐oophorectomy. Histopathological and immunohistochemical examination confirmed the diagnosis of epithelioid trophoblastic tumor. Retrospective assessment revealed mildly elevated serum hCG levels. This case highlights the diagnostic challenge of epithelioid trophoblastic tumor presenting as a hypervascular cervical lesion mimicking uterine arteriovenous malformation. In perimenopausal women with acute vaginal bleeding and AVM‐like imaging findings, ETT should be included in the differential diagnosis to avoid delayed recognition and to guide appropriate perioperative management.

## Graphical abstract

Overview of the intraoperative and gross pathological findings of an epithelioid trophoblastic tumor mimicking uterine arteriovenous malformation.

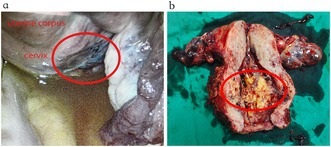

**Key Clinical Message**

Epithelioid trophoblastic tumor should be considered in perimenopausal women presenting with acute vaginal bleeding, low‐level serum hCG elevation, and a hypervascular cervical mass mimicking uterine arteriovenous malformation. Early recognition may prevent misdiagnosis and guide appropriate perioperative management.

## 1 Introduction

Epithelioid trophoblastic tumor (ETT) characterized by amenorrhea and elevated serum human chorionic gonadotropin (hCG) levels with predilection for the lower uterine segment or cervix is the rarest subtype of gestational trophoblastic neoplasia (GTN) accounting for 1.0%–2.0% of all GTN cases [1]. The symptoms of ETT resemble those of other types of gestational trophoblastic disease (GTD) presentation with abnormal vaginal bleeding at the time of diagnosis [2]. Here, we described a perimenopausal woman who presented with sudden profuse vaginal bleeding and cervical mass with low‐level hCG. The patient experienced preoperative uterine artery embolization (UAE) followed by minimally invasive hysterectomy. Post‐operation pathology testified the diagnosis of ETT. We reported the case in hope of providing some useful information for gynecologists, when encountering heavy vaginal bleeding accompanied by cervical space‐occupying lesions, consider the presence of ETT. Given the high risk of hemorrhage associated with hypervascular cervical lesions, perioperative bleeding control may be critical in selected cases.

## 2 Case Presentation

### 2.1 Case History and Examination

A 48‐year‐old Chinese female, G3P2, was admitted in our hospital for suddenly heavy vaginal bleeding for three hours. She reported expelling approximately 300 mL of bright red blood with clots, accompanied by dizziness and a sensation of pelvic pressure. The bleeding occurred after 44 days of amenorrhea. She had a medical history of hepatitis B–related decompensated liver cirrhosis and type 2 diabetes mellitus. Gynecological examination revealed a firm cervical mass measuring approximately 6 cm in diameter, while the uterus was mildly enlarged.

### 2.2 Investigations

**FIGURE 1:**
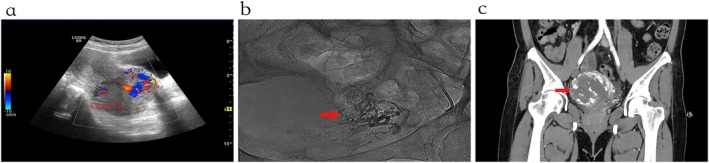
The lesion demonstrated by ultrasonography, abdominal CT and angiograph. a. Heterogeneous mass in the posterior cervical wall with ill‐defined margins and abundant vascular signals. b. Heterogeneous mass in the right posterior cervical wall with multiple enlarged and tortuous vessels on contrast‐enhanced imaging. c. Angiography during uterine artery embolization: Markedly enlarged and tortuous uterine arteries.

ThinPrep cytology (TCT) and human papillomavirus (HPV) tests were negative. Initial laboratory tests showed a hemoglobin level of 114 g/L and a mildly elevated serum human chorionic gonadotropin (hCG) level of 46.04 IU/L, with a negative urine pregnancy test. Transvaginal Color Doppler Ultrasound demonstrated a heterogeneous 7.5 cm × 6.1 cm × 7.0 cm cervical mass with a honeycomb appearance and abundant vascular signals displaying a “color mosaic” pattern, and the possibility of arteriovenous malformation (AVM) cannot be ruled out (Figure 1a). Abdominopelvic CT demonstrated an enlarged uterus with an ill‐defined, heterogeneous mass located in the right posterior of the cervix, approximately 8.5 cm × 7.0 cm × 5.7 cm. Contrast‐enhanced imaging demonstrated multiple enlarged and tortuous vessels within and around the lesion (Figure 1b).

### 2.3 Treatment

**FIGURE 2:**
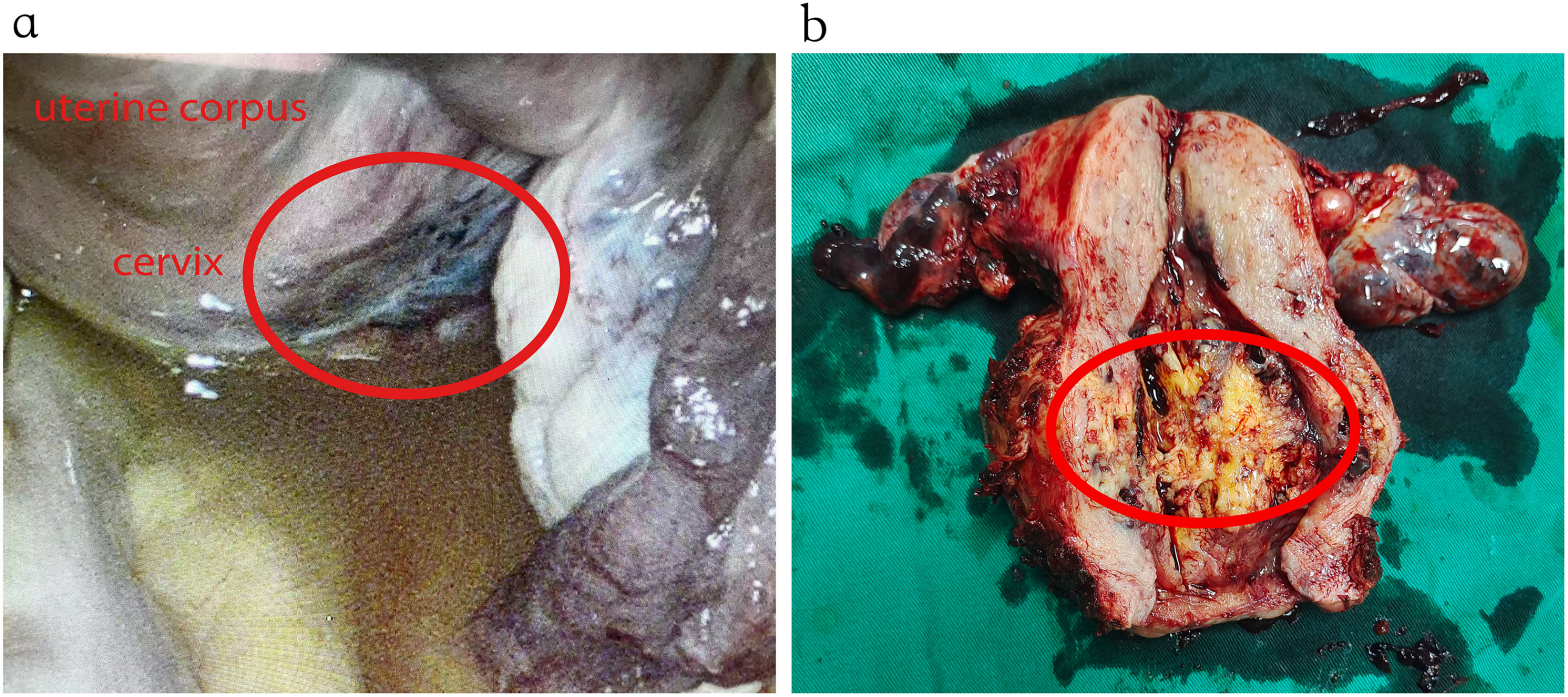
The uterus shown in intraoperative laparoscopy image and postoperative gross examination. a. The markedly thickened cervix protruding into the rectouterine pouch, with abundant, tortuous, and dilated neovascular vessels surrounding the mass. b. Ill‐defined, solid, gray‐yellow cervical tumor with hemorrhagic foci.

During hospitalization, the patient experienced progressive anemia, with hemoglobin levels declining from 114 g/L to 67 g/L within 24 h, indicating ongoing hemorrhage. Given the acute massive vaginal bleeding, progressive hemoglobin decline, hypervascular cervical mass with AVM‐like imaging features, and the patient's limited tolerance to blood loss due to underlying liver cirrhosis, preoperative uterine artery embolization (UAE) (Figure 1c) was performed to reduce the risk of catastrophic intraoperative hemorrhage. Following successful embolization, a laparoscopic total hysterectomy with bilateral salpingo‐oophorectomy was carried out. During the operation, we found the cervix was markedly enlarged (6 cm × 5 cm), firm, and highly vascular, with neovascular connections to the bladder peritoneum (Figure 2a). Gross examination of the uterus after operation demonstrated a 5.5 cm × 5.0 cm × 3.0 cm ill‐defined, solid, gray‐yellow cervical tumor with hemorrhagic foci (Figure 2b).

**FIGURE 3:**
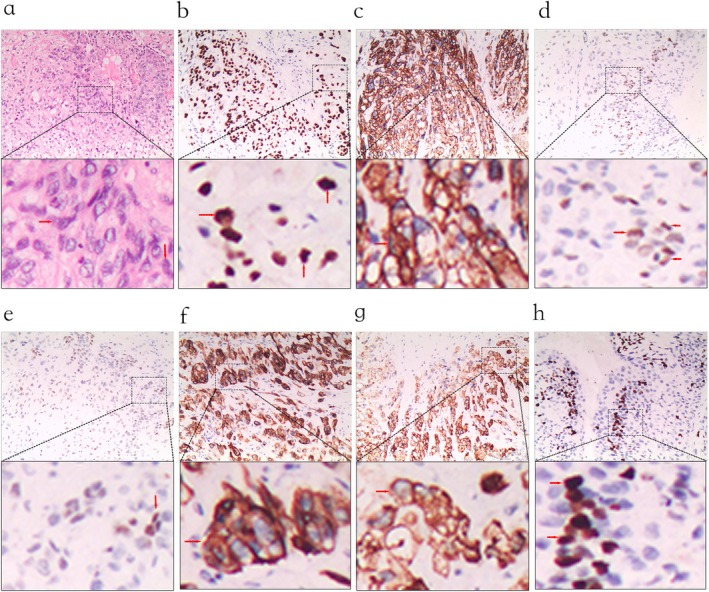
Pathological examination of ETT‐related immune indicators. a. HE examination b. GATA‐3(+) c. pan‐CK(+), d. p40(partial+), e.p63(partial+), f. CK7(+), g.α‐inhibin(+), h.Ki‐67 15% (+).

Histopathological examination demonstrated nests and cords of epithelioid cells with abundant eosinophilic to clear cytoplasm, vesicular nuclei, conspicuous mitoses, and geographic necrosis, with prominent vascular invasion. Immunohistochemistry showed GATA‐3(+), pan‐CK(+), p40(partial+), p63(partial+), CK7(+), α‐inhibin(+), Ki‐67 15% (+), hCG(−), CK20(−). Based on immunological and pathological results, the diagnosis is made as ETT (Figure 3). Retrospective assessment of serum hCG revealed a mild elevation, which, in combination with the pathological and immunohistochemical findings, supported the diagnosis of ETT. We recommended the patient to experience chemotherapy after operation and followed up for 15 months. No recurrent lesions were found, and hCG was 2.42 IU/L.

## 3 Discussion

ETT is a malignant neoplasm of chorionic‐type intermediate trophoblasts, usually arising in the cervix or lower uterine segment, but a few also occurring in the broad ligament, cesarean section scars, lungs, liver, gallbladder, kidney, pancreas, spine, vagina, bladder, and even the colon [2, 3, 4]. The diagnosis of ETT is difficult due to no specificity of signs and symptoms. ETT is usually preceded by normal term pregnancy, rarely by complete or partial mole and spontaneous abortion (16% each) [5]. Patients with ETT performed different clinical presentations which depend on the site of involvement. The majority of patients appeared with abnormal vaginal bleeding, and others complained of amenorrhea, abdominal or back pain, and abdominal bloating [6]. Lesions located in the lungs may experience difficulty breathing or hemoptysis. However, it is sometimes entirely asymptomatic, especially in patients who have extra‐uterine ETT [6]. In our case, the patient complained of heavy vaginal bleeding after amenorrhea.

hCG concentrations are often mildly elevated in ETT (< 2500 IU/L), unlike classic choriocarcinoma with very high hCG values (> 10,000 IU/L) [7], in which accurate measurement of hCG is an important factor to effectively monitor the development of GTD. Notably, mildly elevated serum hCG is also observed in placental site trophoblastic tumor (PSTT) and ectopic pregnancy. In PSTT, hCG levels are frequently < 1000 IU/L (often < 500 IU/L), with reported ranges extending up to 4.5–3480.2 IU/L [8]. In ectopic pregnancy, hCG values commonly fall within the low‐thousands and may remain below the transvaginal ultrasound discriminatory zone reported in the literature (~1000–3500 IU/L), with a subset of cases presenting with hCG < 1000 IU/L [9]. Therefore, in retrospect, the low‐level hCG elevation, together with cervical localization and characteristic histopathological and immunophenotypic findings, collectively supported the diagnosis of epithelioid trophoblastic tumor. Nevertheless, high levels of hyperglycosylated hCG have been reported to correlate with GTN, whereas the β subunit of hCG has been used to help discriminate PSTT and ETT from other forms of GTN [10]. Though hCG is a less reliable marker for the diagnosis and follow‐up of the development of ETT, we still observed a mild increase in hCG and used it for postoperative monitoring.

Although ultrasonography plays a central role in the initial evaluation and differential diagnosis of gestational trophoblastic neoplasia, there are no direct ultrasonographic signs to differentiate ETT from other cervical lesions. Conventional grayscale and color Doppler ultrasound are often the first‐line imaging modalities for assessing lesion location, growth pattern, and vascular architecture [11]. However, overlap in sonographic features among different GTN subtypes may complicate preoperative diagnosis. It is reported that the manifestation of ETT is a well‐circumscribed tumor border surrounded by a hypoechogenic halo, growing in an expansive fashion and invading the cervix or myometrium deeply [12]. In this patient, transvaginal color Doppler ultrasound demonstrated abundant vascular signals with low resistance within and around the cervix. Plain CT demonstrated a heterogeneous mass in the right posterior cervix, and contrast‐enhanced CT revealed multiple enlarged and tortuous vessels within and around the lesion, resembling the imaging features of AVM [13], which further increased the difficulty in making an accurate diagnosis. Recent evidence suggests that uterine artery Doppler parameters may provide additional diagnostic value. In a prospective cohort study, Cioffi et al. demonstrated that placental site trophoblastic tumor (PSTT) is associated with a significantly reduced uterine artery pulsatility index, reflecting its highly vascular and invasive nature, and that Doppler assessment may aid in differentiating PSTT from other GTN subtypes [14]. Although similar Doppler‐based criteria have not yet been established for epithelioid trophoblastic tumor, this finding highlights the potential utility of hemodynamic evaluation in trophoblastic disease.

The differential diagnosis of ETT primarily includes PSTT, choriocarcinoma (CC), and cervical carcinoma, which may share overlapping clinical or histological features. From an immunohistochemical perspective, PSTT typically shows diffuse expression of human placental lactogen (hPL) and hCG, while p63 and p40 are usually negative, reflecting its origin from implantation‐site intermediate trophoblasts [15, 16]. In contrast, ETT characteristically demonstrates positivity for p63 and/or p40, epithelial markers such as cytokeratins, and GATA3, with absent or weak hCG expression [15, 16, 17]. CC is distinguished by a biphasic population of cytotrophoblasts and syncytiotrophoblasts and shows strong, diffuse hCG expression with a markedly elevated proliferation index [18]. Cervical carcinoma may also enter the differential diagnosis due to p63 and p40 positivity; however, it typically demonstrates diffuse CK5/6 and p16 overexpression related to HPV infection [19].

Hysterectomy is the primary method to treat ETT, especially for metastatic disease. In our case, radiological features closely resembled those of AVM, a condition associated with a substantial risk of catastrophic intraoperative bleeding [20]. In addition, the patient's underlying liver cirrhosis further limited her physiological tolerance to blood loss. Alternative management strategies, including immediate surgical intervention without embolization or conservative medical management, were considered less appropriate in this context. Given the active bleeding, progressive anemia, and strong suspicion of AVM based on imaging, proceeding directly to surgery without vascular control was deemed unsafe. Taken together, these factors supported the decision to perform preoperative UAE to stabilize the patient and reduce perioperative hemorrhagic risk [21]. To date, UAE in the management of ETT has rarely been discussed in the literature. The successful perioperative course in this case suggests that preoperative UAE may represent a feasible adjunctive option in selected patients with hypervascular cervical ETT. Given the rarity of ETT and the limited available data, this observation should be interpreted with caution. Further accumulation of cases and systematic evaluation are required to clarify the role, indications, and safety of prophylactic UAE in the management of ETT.

With the trend of tumors occurring at a younger age, treatments that preserve fertility are becoming increasingly important. Fertility‐preserving treatment in ETT is rarely reported, with only about 10 cases documented [22, 23]. Liu et al. reported five ETT patients in stage I: one achieved successful cesarean delivery, one developed lung metastasis and died after chemotherapy alone, and the remaining three showed no recurrence or death at last follow‐up [22]. In addition, for patients of reproductive age who are required to undergo hysterectomy or high‐dose chemotherapy, fertility preservation can be achieved through oocyte or embryo freezing [24]. The use of gonadotropin‐releasing hormone agonists and antagonists has further improved these techniques by protecting ovarian function during chemotherapy [25]. Other promising developments include in vitro maturation of oocytes and ovarian tissue cryopreservation. The studies have shown that these procedures help preserve fertility without compromising the success or outcome of cancer treatment [25]. Reproductive counseling should be initiated promptly after cancer diagnosis and staging in young patients, allowing adequate time to discuss and select the most appropriate fertility preservation options based on both oncological and reproductive prognoses.

ETT is generally resistant to chemotherapy, and chemotherapy alone is insufficient to cure advanced, metastatic, or locally advanced disease [22]. Nevertheless, it continues to play an important role in the management of ETT when surgical treatment is not feasible or effective. For patients with chemotherapy‐resistant disease, newer treatments like immune checkpoint inhibitors (ICIs) have shown promise in achieving complete remission. Pembrolizumab has shown promise in treating chemo‐resistant ETT, as evidenced by multiple case reports [26]. These findings stress the need for further research to optimize the use of ICIs in ETT.

Given the pivotal role of the tumor microenvironment (TME) in determining the response to ICIs, accurate characterization of TME composition has become increasingly important. The presence of tumor‐infiltrating lymphocytes (TILs), particularly CD8+ cytotoxic T cells, correlates with better responses to ICIs [27]. In this context, the rapid expansion of clinical, imaging, molecular, and immunological data has created opportunities for the application of artificial intelligence (AI)–based approaches [28]. AI‐driven tools can integrate complex and heterogeneous datasets, identify predictive patterns that are difficult to detect using conventional methods, and support individualized risk stratification and treatment decision‐making [28]. Such approaches may be particularly valuable for predicting immunotherapy responsiveness in rare tumors such as ETT.

In summary, ETT should be considered in perimenopausal women with acute vaginal bleeding, mildly elevated serum hCG, and hypervascular cervical lesions mimicking uterine arteriovenous malformation. This case emphasizes the importance of diagnostic vigilance and suggests that preoperative uterine artery embolization may be a useful adjunct to reduce perioperative hemorrhagic risk in selected patients.

## Author Contributions

**Huihua Xiang:** conceptualization, data curation, formal analysis, methodology, writing – original draft, writing – review and editing. **Xiaoxiao Feng:** project administration, validation, visualization. **Shanrong Shu:** funding acquisition, resources, supervision.

## Funding

The authors have nothing to report.

## Consent

Written informed consent was obtained from the patient for publication of this case report and any accompanying images.

## Conflicts of Interest

The authors declare no conflicts of interest.

## Data Availability

Data sharing is not applicable to this article as no datasets were generated or analyzed during the current study.

## References

[1] F. Gorun , L. Tomescu , A. Motoc , et al., “Clinical Features and Management of Trophoblastic Epithelioid Tumors: A Systematic Review,” Medicine (Baltimore) 101, no. 30 (2022): e29934, 10.1097/md.0000000000029934. 35905248 PMC9333520

[2] S. Radhakrishnan , N. N. Suvarna , S. Sreeram , and S. Bhat , ““Colon” Ised by the Unexpected: A Case of Extrauterine Epithelioid Trophoblastic Tumour,” Diagnostic Pathology 20, no. 1 (2025): 61, 10.1186/s13000-025-01617-2. 40413529 PMC12102896

[3] R. Aničić , A. Rakić , R. Maglić , et al., “A Rare Case of Epithelioid Trophoblastic Tumor Presenting as Hematoma of a Caesarean Scar in the Lower Uterine Segment,” Medicina (Kaunas, Lithuania) 58, no. 1 (2021): 34, 10.3390/medicina58010034. PMC8777674 35056342

[4] J. Gallardo , K. Hummel , H. Siatecka , et al., “Epithelioid Trophoblastic Tumor Presenting as an Adnexal Mass: Report of a Diagnostically Challenging Case,” International Journal of Surgical Pathology 31, no. 5 (2023): 651–655, 10.1177/10668969221117983. 35946122

[5] T. Chawla , G. Bouchard‐Fortier , G. Turashvili , R. Osborne , K. Hack , and P. Glanc , “Gestational Trophoblastic Disease: An Update,” Abdominal Radiology 48, no. 5 (2023): 1793–1815, 10.1007/s00261-023-03820-5. 36763119

[6] J. Li , Z. Du , T. Xu , C. Li , S. Ba , and H. Zhu , “Epithelioid Trophoblastic Tumor With Lung Metastasis: A Case Report and Literature Review,” Medicine (Baltimore) 103, no. 27 (2024): e38108, 10.1097/md.0000000000038108. 38968534 PMC11224836

[7] M. M. Frijstein , C. A. R. Lok , N. E. van Trommel , et al., “Management and Prognostic Factors of Epithelioid Trophoblastic Tumors: Results From the International Society for the Study of Trophoblastic Diseases Database,” Gynecologic Oncology 152, no. 2 (2019): 361–367, 10.1016/j.ygyno.2018.11.015. 30473257

[8] Y. Zhou , H. Lu , C. Yu , Q. Tian , and W. Lu , “Sonographic Characteristics of Placental Site Trophoblastic Tumor,” Ultrasound in Obstetrics & Gynecology 41, no. 6 (2013): 679–684, 10.1002/uog.12269. 22807194

[9] Q. Lu , Y. Wang , X. Sun , et al., “The Diagnostic Role of the β‐hCG Discriminatory Zone Combined With the Endometrial Pattern for Ectopic Pregnancy in Chinese Women,” Scientific Reports 9, no. 1 (2019): 13781, 10.1038/s41598-019-50151-x. 31551446 PMC6760119

[10] F. E. M. Froeling , R. Ramaswami , P. Papanastasopoulos , et al., “Intensified Therapies Improve Survival and Identification of Novel Prognostic Factors for Placental‐Site and Epithelioid Trophoblastic Tumours,” British Journal of Cancer 120, no. 6 (2019): 587–594, 10.1038/s41416-019-0402-0. 30792530 PMC6461960

[11] P. Cavoretto , R. Cioffi , G. Mangili , et al., “A Pictorial Ultrasound Essay of Gestational Trophoblastic Disease,” Journal of Ultrasound in Medicine 39, no. 3 (2020): 597–613, 10.1002/jum.15119. 31468566

[12] Y. Zhu , G. N. Zhang , R. B. Zhang , Y. Shi , D. F. Wang , and R. He , “Sonographic Image of Cervix Epithelioid Trophoblastic Tumor Coexisting With Mucinous Adenocarcinoma in a Postmenopausal Woman: A Case Report,” Medicine (Baltimore) 96, no. 38 (2017): e7731, 10.1097/md.0000000000007731. 28930821 PMC5617688

[13] S. Chen , X. Song , J. Su , et al., “Uterine Arteriovenous Fistula: A Case Report and Literature Review,” Medicine (Baltimore) 104, no. 31 (2025): e43549, 10.1097/md.0000000000043549. 40760517 PMC12324039

[14] R. Cioffi , P. I. Cavoretto , G. Sabetta , et al., “Additional Value of Uterine Artery Doppler Pulsatility Index for Ultrasound Diagnosis of Placental Site Trophoblastic Tumor: Prospective Cohort Study,” Ultrasound in Obstetrics & Gynecology 66, no. 1 (2025): 73–80, 10.1002/uog.29235. 40387112 PMC12209685

[15] I. M. Shih and R. J. Kurman , “Epithelioid Trophoblastic Tumor: A Neoplasm Distinct From Choriocarcinoma and Placental Site Trophoblastic Tumor Simulating Carcinoma,” American Journal of Surgical Pathology 22, no. 11 (1998): 1393–1403, 10.1097/00000478-199811000-00010. 9808132

[16] N. Buza , “Gestational Trophoblastic Disease: Contemporary Diagnostic Approach,” Surgical Pathology Clinics 15, no. 2 (2022): 197–218, 10.1016/j.path.2022.02.002. 35715158

[17] B. Kaur , “Pathology of Gestational Trophoblastic Disease (GTD),” Hematology/Oncology Clinics of North America 38, no. 6 (2024): 1191–1217, 10.1016/j.hoc.2024.08.017. 39322461

[18] M. J. Seckl , N. J. Sebire , and R. S. Berkowitz , “Gestational Trophoblastic Disease,” Lancet 376, no. 9742 (2010): 717–729. 20673583 10.1016/S0140-6736(10)60280-2

[19] T. M. Darragh , T. J. Colgan , J. T. Cox , et al., “The Lower Anogenital Squamous Terminology Standardization Project for HPV‐Associated Lesions: Background and Consensus Recommendations From the College of American Pathologists and the American Society for Colposcopy and Cervical Pathology,” Archives of Pathology & Laboratory Medicine 136, no. 10 (2012): 1266–1297, 10.5858/arpa.LGT200570. 22742517

[20] D. Sridhar and R. L. Vogelzang , “Diagnosis and Treatment of Uterine and Pelvic Arteriovenous Malformations,” Endovascular Today 17, no. 1 (2018): 73.

[21] G. M. Salazar , J. C. Petrozza , and T. G. Walker , “Transcatheter Endovascular Techniques for Management of Obstetrical and Gynecologic Emergencies,” Techniques in Vascular and Interventional Radiology 12, no. 2 (2009): 139–147, 10.1053/j.tvir.2009.08.007. 19853231

[22] W. Liu , J. Zhou , J. Yang , and X. Huang , “A Multicenter Retrospective Study of Epithelioid Trophoblastic Tumors to Identify the Outcomes, Prognostic Factors, and Therapeutic Strategies,” Frontiers in Oncology 12 (2022): 907045, 10.3389/fonc.2022.907045. 35677151 PMC9169038

[23] Z. Huang , Y. Yu , D. Wen , N. Wang , and L. Zeng , “The Fertility‐Sparing Treatment and Outcome of Epithelioid Trophoblastic Tumor Isolated to Lung: A Case Report and Review Literature,” Frontiers in Oncology 14 (2024): 1337213, 10.3389/fonc.2024.1337213. 38549926 PMC10972860

[24] G. Gullo , M. Satullo , E. Conti , et al., “Gestational Trophoblastic Disease: Diagnostic and Therapeutic Updates in Light of Recent Evidence: A Literature Review,” Medicina (Kaunas, Lithuania) 61, no. 9 (2025): 1–17, 10.3390/medicine61091642. PMC12471455 41011032

[25] L. De Paola , G. Napoletano , G. Gullo , F. Circosta , G. Montanari Vergallo , and S. Marinelli , “The Era of Increasing Cancer Survivorship: Trends in Fertility Preservation, Medico‐Legal Implications, and Ethical Challenges,” Open Med (Wars) 20, no. 1 (2025): 20251144, 10.1515/med-2025-1144. 39958979 PMC11826245

[26] J. Zeng , J. Zhang , J. Wang , L. Xu , C. Wang , and R. Yin , “Immunotherapy in Gestational Trophoblastic Neoplasia: Advances and Future Directions,” Frontiers in Immunology 16 (2025): 1544585, 10.3389/fimmu.2025.1544585. 40292281 PMC12021912

[27] L. A. Diaz, Jr. , K. K. Shiu , T. W. Kim , et al., “Pembrolizumab Versus Chemotherapy for Microsatellite Instability‐High or Mismatch Repair‐Deficient Metastatic Colorectal Cancer (KEYNOTE‐177): Final Analysis of a Randomised, Open‐Label, Phase 3 Study,” Lancet Oncology 23, no. 5 (2022): 659–670, 10.1016/s1470-2045(22)00197-8. 35427471 PMC9533375

[28] E. Chitoran , V. Rotaru , A. Gelal , et al., “Using Artificial Intelligence to Develop Clinical Decision Support Systems‐The Evolving Road of Personalized Oncologic Therapy,” Diagnostics (Basel, Switzerland) 15, no. 18 (2025): 129–138, 10.3390/diagnostics15182391. 41008762 PMC12468058

